# Tibial Malrotation Following Intramedullary Nailing: A Literature Review

**DOI:** 10.7759/cureus.19683

**Published:** 2021-11-17

**Authors:** André R Coelho Fernandes, Karanjeet S Sagoo, Jennifer Oluku, Kamalpreet S Cheema

**Affiliations:** 1 Trauma and Orthopaedic Surgery, Lewisham and Greenwich NHS Trust, London, GBR; 2 Trauma and Orthopaedics, Kings College London, London, GBR; 3 Radiology, Guy's and St Thomas' NHS Foundation Trust, London, GBR; 4 Trauma and Orthopaedic Surgery, Guy's and St Thomas' NHS Foundation Trust, London, GBR

**Keywords:** orthopaedics trauma, tibia, tibia diaphysis, rotation, literature review of disease, malaligment, tibia nail, tibia shaft fracture, s: intramedullary nail, malrotation

## Abstract

The use of intramedullary nail fixation remains the operation of choice for managing unstable and displaced tibia diaphyseal fractures. The literature shows that although commonly performed, there is not a standard approach when performing intramedullary nailing of the tibia; it could be hypothesised that this lack of standardisation may be contributing to the noted complications. This systematic review will look into intramedullary nailing of the tibia in all its parts, from identification of patients through to the surgical procedure techniques and finally the intra- and post-operative complications.

A systematic review was conducted using the Preferred Reporting Items for Systematic Reviews and Meta-Analyses (PRISMA) guidelines. Search terms included “tibial intramedullary nail” OR “tibial intramedullary rod” OR “tibial IM nail” OR “tibial interlock” AND “malrotation”, and “tibial intramedullary nailing” OR “tibial im nail” OR “tibial interlock” OR “tibial rod” AND “malrotation”. Two independent reviewers conducted searches in PubMed, OvidSP for Medline and Embase as well as Cochrane Library using the same search strategy. Searches were conducted on 20 January 2021. Any disagreements were resolved by discussion with a third independent reviewer.

This systematic review revealed there are gaps in the literature and in the management process of these patients, and suggested that a systematic approach using ‘Get It Right First Time’ (GIRFT), intraoperative assessment, validated assessment tools, and imaging postoperatively should be used to improve outcomes. Following the use of this framework, it is hoped that the incidence of malrotation post tibia intramedullary nailing will be reduced, however, it is acknowledged that more high evidence studies need to be carried out and further done to optimise the care of these patients.

## Introduction and background

Introduction

The use of intramedullary nail fixation remains the operation of choice for managing unstable, and displaced tibia diaphyseal fractures. Intramedullary nailing aims to: aid bony union, restore length, alignment, and rotation of the fractured tibia; it has added advantages as it allows minimal surgical dissection - preserving the blood supply to the fracture site, and the implant also acts as a load-sharing device - giving biomechanical fracture stabilisation and allowing early patient mobilisation. No surgical procedure is without complication and intramedullary nailing is not exempt; high incidences of malrotation and malunion have been reported amongst others.

The literature shows that although commonly performed, there is no standard approach when performing intramedullary nailing of the tibia; it could be hypothesised that this lack of standardisation may be contributing to the noted complications. Malrotation, in particular, has a high impact physically and psychologically on the patient, making it an important complication to address in improving patient outcomes. By reviewing practice and the literature an optimal and standardised approach can be sought in managing these subsets of patients, but more importantly, reducing the incidence of the complication occurring.

This systematic review will look into intramedullary nailing of the tibia in all its parts, from identification of patients through to the surgical procedure techniques and finally the intra- and postoperative complications. In addition, this review aims to answer:

1. Can we improve our intraoperative technique to ensure alignment and rotation?

2. What is the best method to assess malrotation postoperatively?

3. How can we manage malrotation successfully and reduce morbidity?

After analysis, this review aims to suggest a systematic and standardized approach to managing patients with malrotation and to reduce its incidence.

Background

Tibial shaft fractures are the commonest long-bone fracture in the body, usually as a result of high impact forces. It commonly occurs in high-energy collisions for example road traffic accidents, falls from a height, or twisting motions that can occur in high impact/contact sports. Intramedullary nail fixation is a common and popular fixation method in managing these injuries.

Usually performed as a closed operative technique, an accurate reduction is dependent on appropriate radiographic imaging and clinical examination, however, even with these interventions, the incidence of malrotation has been reported as high as 30% in practice [1-3].

Tibial axial rotational malalignment can be defined as the twisting of the proximal and distal ends of the bone on the same axis. It can often be overlooked on reductions which can bring about serious functional repercussions. Lower extremity rotational malalignment in the lower extremities can cause changes in the biomechanics of the joints above and below - from simple cosmetic problems to severe functional impairment, this can lead to a significant deterioration in the patient’s quality of life. Clementz et al discussed that a wide range of normal existed in tibia rotation and torsion placements, with external rotation of the right tibia and the difference in torsion between the right and left tibia ranging from -11 to 15 degrees in normal adults [4]. This high degree of variability of tibial torsion plays a factor in making it technically more challenging and difficult to assess for rotational alignment postoperatively.

## Review

Search strategies

**Figure 1:**
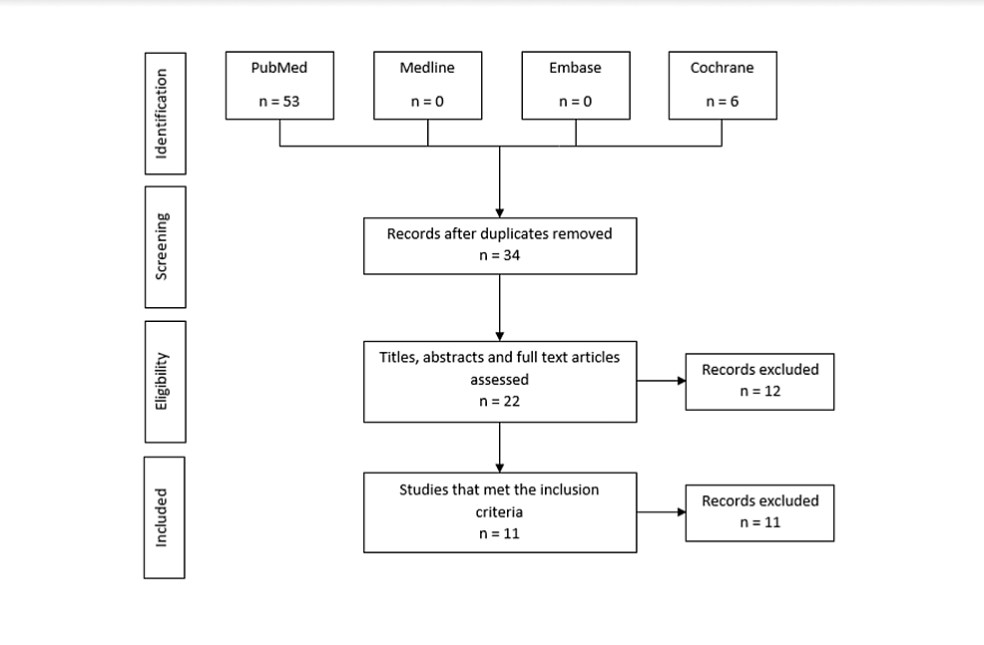
PRISMA literature search methodology PRISMA - Preferred Reporting Items for Systematic Reviews and Meta-Analyses

A systematic review was conducted using the Preferred Reporting Items for Systematic Reviews and Meta-Analyses (PRISMA) guidelines (Figure 1). Search terms included “tibial intramedullary nail” OR “tibial intramedullary rod” OR “tibial IM nail” OR “tibial interlock” AND “malrotation”, and “tibial intramedullary nailing” OR “tibial im nail” OR “tibial interlock” OR “tibial rod” AND “malrotation”. Two independent reviewers conducted searches in PubMed, OvidSP for Medline and Embase as well as Cochrane Library using the same search strategy. Searches were conducted on 20 January 2021. Any disagreements were resolved by discussion with a third independent reviewer.

Inclusion criteria

Inclusion criteria included studies published in English, tibial fracture fixation using intramedullary nailing. This included fixation for both open and closed tibial fractures. All fracture morphologies from simple two-part tibial fracture to comminuted fractures as well as intra- and extra-articular fractures were considered. Study types included were Randomised control trials, meta-analyses and case reports were also included in our analysis.

Outcome measures

Primary outcomes of interest consisted of incidence and degrees of tibial malrotation. Secondary outcomes of interest consisted of methods of determining malrotation (postoperative CT or clinical examination), postoperative function, postoperative complications (i.e. wound issues), time to union and further procedures.

Results

The literature search identified a total of 59 studies. Following the removal of 25 duplicates, the remaining 34 studies were analysed according to their title, abstract, and full text, following which 12 were excluded. The remaining studies were analysed to ensure they meet the inclusion criteria, of which 11 studies were selected. Studies included case reports, case series, systematic and literature reviews [2-3].

Included Studies

-2002 Kevin M. Kahn, Rodney K. Beals [1] 

-2004 Puloski S, Romano C, Buckley R et al [2] 

-2021 Theriault B, Turgeon AF, Pelet S et al [3]

-1988 Clementz BG [4]

-2013 Manish Prasad, Sanjay Yadav, Ajaydeep Sud et al [5]

-2014 Ferhat Say, Murat Bülbül [6]

-2014 Jialiang Guo, Yingze Zhang, Zhiyong Hou et al [7]

-2016 Sher Baz Khan, Yasir Mohib et al [8]

-2018 Adel Ebrahimpour Jafarinejad, Hooman Bakhshi et al [9]

-2018 Kyohei Takase et al [10]

-2018 Fatih Inci, Ahmet Ozgur Yildirim et al [11]

-2019 Emre AnJl Özbek et al [12]

Eleven studies were considered in the final analysis, with a total number of 425 fractures (n=429, mean ± 95% CI=39 (21.2-56.8)). All included patients were treated with tibial intramedullary nail devices, and 110 showed some degree of malrotation postoperatively (n=110, mean ± 95% CI=10.1 (4.26-15.9)). Expressed as a percentage, a mean of 34.1% of patients had malrotation (mean ± 95% CI: 34.1 (15.8-52))

In all studies, malrotation was established as x>10 degrees in both internal and external rotation with the exception of Khan S et al [7], who considered up to 15 degrees of external rotation as acceptable (Table 1). In total, there were three hundred closed fractures and 55 open fractures (Theriault et al [3] did not disclose the type of fracture in their 70 patients).

**Table 1:** Statistical Representation Tibial Diaphyseal Fractures included in this review. VARS.P - Variance, population VARS.S - Variance, sample

Values	Total	Malrotation	%Malrotation
Mean	39	10.091	34.10
Standard Error	8	2.616	8.20
Median	40	8	24.7
Mode	9	5	19
Standard Deviation	26.533	8.677	27.20
Sample Variance	704	75.29	740.06
Kurtosis	-1.0772	0.83	2.82
Skewness	0.068154	1.22	1.68
Range	80	28	94
Minimum	1	1	6
Maximum	81	29	100
Sum	429	111	375.2
Count	11	11	11
Confidence Level (95%)	17.82	5.82	18.28
VAR.P	640	68.45	672.79
VAR.S	704	75.29	740.06
Mean +- 95% Cl: 39	21.2-56.8	4.26-15.9	15.8-52.4

Fracture complexity

The literature shows that the higher the energy, degree of comminution, and displacement of the fragments, the higher the chance of post-nailing axial malrotation. In contrast, the presence of an intact fibula was a consistent preventing factor of rotational malalignment (Tables 2, 3). Khan et al and Prasad et al displayed that the higher the degree of comminution the higher the chances of malrotation postoperatively [4, 7].

**Table 2:** Comparison between Prasad et al (2013) and Khan et al (2016) studies

	Population	Simple	Wedged	Complex	Malrotation
2013 Prasad et al [4]	60	56	4	0	10
2016 Khan et al [7]	81	52	11	18	20

**Table 3:** Descriptive table with the fracture pattern and malrotation incidence across all included studies

Study ID	No Participants	Patients w/ Malrotation	Degrees of Malrotation	Simple	Wedge	Complex	Closed	Open
2002 Kevin M Kahn, Rodney K. Beals [1]	3	2 (66%)	x>15°	2	1	0	3	0
2004 S Puloski et al [2]	22	8 (36%)	x>10°	13	7	2	18	4
2012 Benoit Theriault et al [3]	70	29 (41%)	x>10°	N/A	N/A	N/A	N/A	N/A
2013 Manish Prasad et al [4]	60	10 ( 6%)	x>10°	56	4	0	36	24
2014 Ferhat Say, Murat Bulbul [5]	26	5 (19%)	x>10°	14	9	3	22	4
2014 Jialiang Guo, et al [6]	24	5 (21%)	x>10°	24	0	0	20	4
2016 Sher Baz Khan et al [7]	81	20 (24.7%)	x>10° (Int.Rotation) x>15° (Ext.Rotation)	52	11	18	81	0
2018 Adel Ebrahimpour Jafarinejad et al [8]	60	18 (30%)	x>10°	30	21	9	60	0
2018 Kyohey Takase et al [9]	1	1 (100%)	25°	1	0	0	1	0
2018 Fatih Inci et al [10]	42	8 (19%)	x>10°	25	17	0	37	5
2019 Emre Anjl Özbek et al [11]	40	5 (12.5%)	x>10°	26	13	1	26	14

Intraoperative methods to ensure rotation

Different ways of assessing intraoperative rotation have been described in the literature: one includes assessing intraoperative radiographs and comparing with the contralateral limb; values are attained by drawing lines on the transcondylar axis of the femur proximally and the tangent to the articular surface of the medial malleolus distally. There are limited reports of its use, and it is thought that intraoperative fluoroscopy of the contralateral limb can be technically challenging and unreliable [1].

Inci et al assessed rotational alignment with MRI imaging: their study showed that the mean delta difference between their study groups (external rotation tibial apparatus [ERTA] 3,8° vs Clinical 8.1°) was statistically significant. They concluded that an ERTA should be the mainstay of tibia intramedullary nailing surgery.

Postoperative investigations 

With the array of methods to evaluate postoperative malrotation, from clinical methods to the use of cross-sectional imaging, it was found that the preference was for cross-sectional imaging, with CT imaging being the most prefered (58.3% of the studies).

**Figure 2:**
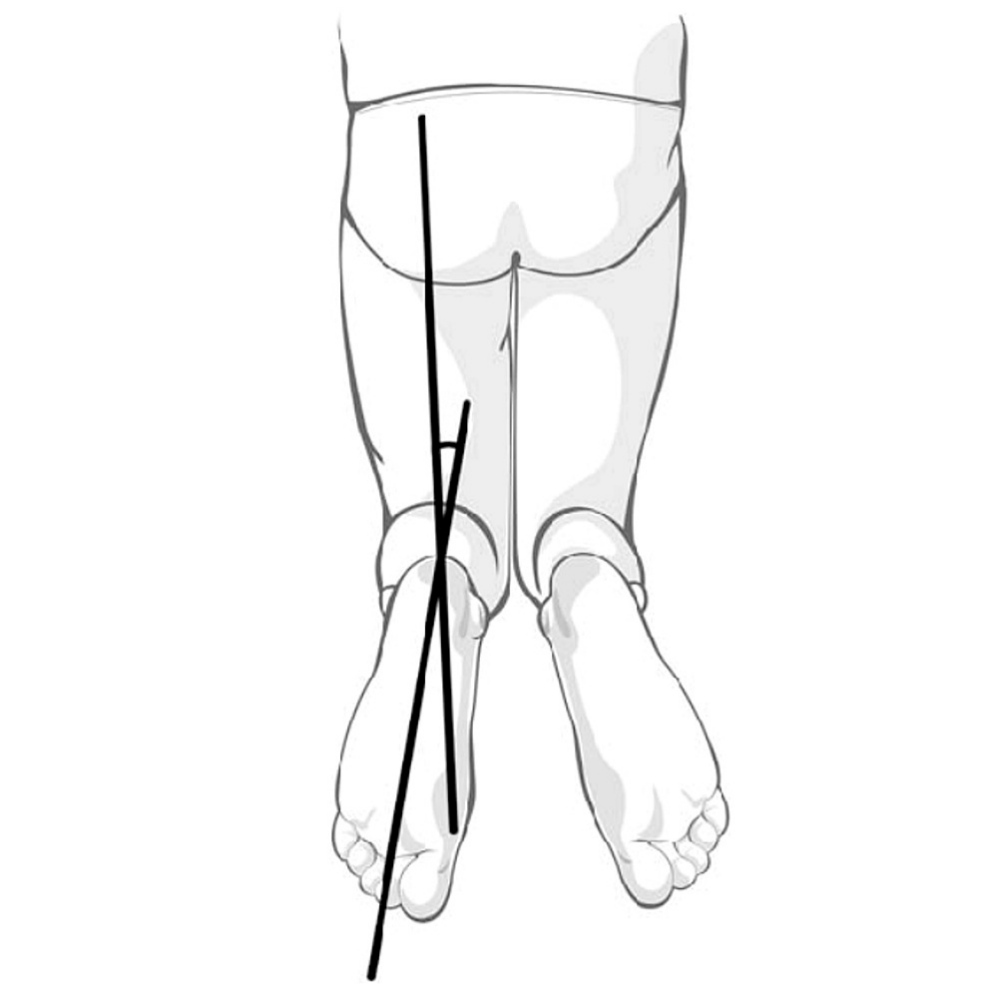
Clinical assessment of tibial malalignment (diagram)

In clinical assessment, the most commonly used technique is the measurement of the foot-thigh angle (Figure 2). The operator compares the measurements between the longitudinal axis of the thigh with the longitudinal axis of the foot. This can be performed with the patient supine or prone, however, the latter is most commonly prefered. In prone, the patient should have their knee flexed to 90 degrees and the ankle at neutral flexion (Video 1).

**Video 1:** Visual representation of the tibial torsion thigh-foot angle test

The clinical assessment comes with its disadvantages as it is prone to operator- and patient-dependent factors (i.e., operator experience or patient’s frailty).

Say et al [5] describe CT as a superior method of imaging (Table 4), with good sensitivity, particularly at lower malrotation values, and not subject to observer variability - no significant difference between reporters whereas an examiner might claim malrotation as nonexistent.

**Table 4:** Postoperative malalignment assessment tools of choice

CT	MRI	Clinical *OE*	Intraop Fluoroscopy
2002 Kevin M Kahn, Rodney K. Beals [1]	2018 Fatih Inci, Ahmet Ozgur Yildirim [10]	2012 Benoit Theriault, Alexis F. Turgeon [3]	2016 Sher Baz Khan, Yasir Mohib [7]
2004 S Puloski, C. Romano [2]		2013 Manish Prasad, Sanjay Yadav, Ajaydeep Sud [4]	
2012 Benoit Theriault, Alexis F. Turgeon [3]		2019 Emre Anjl Özbek [11]	
2014 Ferhat Say, Murat Bulbul [5]			
2014 Jialiang Guo, Yingze Zhang, Zhiyong Hou [6]			
2018 Adel Ebrahimpour Jafarinejad, Hooman Bakhshi [8]

 ‘The difference between the mean clinical thigh-foot angle (TFA) measurement (1.1 ± 5.6) and the mean CT measurement (4.78 ± 9.5) was statistically significant (p\0.001, Wilcoxon paired-sample test). No statistically significant relationship was determined between a rotational difference over 108 and the AO fracture type, fracture location, and fibula fixation’ [6].

CT imaging does come with its own disadvantages, including cost, availability (in austere areas) and ionising radiation to the patient.

Revision surgery

Three out of 110 cases reviewed (2.73%) required a form of revision surgery. Two cases involved removal of the nail with osteotomy, and one underwent readjustment of the nail (distal locking screws reinserted in a new position) with a tibial/fibula osteotomy.

A few techniques are described in the literature on how to approach malrotation requiring operative management; however, there is insufficient data available, with no studies comparing outcomes from the methods described below [3].

- Removal of Nail +- Proximal/Distal Derotational Osteotomy + Locked IMN insertion

- Removal of Nail + Supramalleolar Derotational Osteotomy + Plate Fixation

- Removal of Nail + Osteotomy + Ilizarov Ex Fix (Lengthening + Rotational Correction)

Functional outcome

Out of the 12 studies reviewed, only two evaluated the functional outcome of patients, Theriault et al and Özbek et al [3, 11].

There are a few methods described as to how to systematically assess the functional outcome of the lower limb after surgery. These methods will be briefly described in this section. 

The Lower Extremity Functional Scale (LEFS) defined by Theriault et al was the most commonly used method found in all the studies; it was found to be a comprehensive, systematic, and validated tool for the assessment of the knee or the ankle joint - the two articulations most likely to be affected by tibial malrotation [3].

Theriault’s findings also showed that in all patients with over 10 degrees of tibial malrotation following locked intramedullary nailing there was no significant difference, either clinically or statistically, inferring the use of locking screws helped in adding positive functional outcomes.

The mean LEFS score was 70.8 points in the malrotation group, compared with 72.6 points in the normal rotation group, for a mean difference of 1.8 points, which was not significant (p = 0.41) or clinically important (<9 points). The results of the Olerud-Molander score (p = 0.18) and the six-minute walk test (p = 0.38) were not significantly different between the two groups’ (Table 5)

**Table 5:** Functional outcomes according to higher degrees of tibial malrotation Source: Theriault et al [3] LEFS - Lower Extremity Functional Scale

	Tibia Rotation ≥ 15 degrees			Tibial Rotation ≥ 20 degrees		
Variable	Malrotation (N=12)	No Malrotation (N=58)	P Value	Malrotation (N=6)	No Malrotation (N=64)	P Value
LEFS (points)	69.3 ± 8.9	72.3 ± 8.6	0.32	69.2 ± 8.5	72.1 ± 8.7	0.26
Olerud-Molander (points)	75.4 ± 18.3	81.5 ±19.8	0.34	75.0 ± 22.2	80.9 ± 19.4	0.39
Six Minute Walk Test (meters)	579.5 ± 80.8	575.7 ± 83.3	0.89	598.5 ± 46.5	574.2 ± 85.3	0.38

Lower limb function assessment tools

Lower Extremity Functional Scale (LEFS):

The LEFS englobes the sum of 20 different activities scoring up to a maximum of 80 points. The lower the score the greater the disability. The minimal detectable change (MDC) is 9 scale points. The minimal clinically important difference (MCID) is 9 scale points. Percent of maximal function = (LEFS score) / 80 * 100 (Table 6) [13].

**Table 6:** The Lower Extremity Functional Scale Source: Binkley et al [13]

Activities	Extreme Difficulty or Unable	Quite a bit of difficulty	A little bit of difficulty	No difficulty
Any of your usual work, housework or school activities.	0	1	2	3
Your usual hobbies, recreational or sporting activities.	0	1	2	3
Getting into or out of the bath.	0	1	2	3
Walking between rooms.	0	1	2	3
Putting on your shoes or socks.	0	1	2	3
Squatting.	0	1	2	3
Lifting an object, like a bag of groceries from the floor.	0	1	2	3
Performing light activities around your home.	0	1	2	3
Performing heavy activities around your home.	0	1	2	3
Getting into or out of a car.	0	1	2	3
Walking 2 blocks.	0	1	2	3
Walking a mile.	0	1	2	3
Going up or down 10 stairs (about 1 flight of stairs).	0	1	2	3
Standing for 1 hour.	0	1	2	3
Sitting for 1 hour.	0	1	2	3
Running on even ground.	0	1	2	3
Running on uneven ground.	0	1	2	3
Making sharp turns while running fast.	0	1	2	3
Hopping.	0	1	2	3
Rolling over in bed.	0	1	2	3

Tegner Lysholm Knee Scoring Scale: 

The Tegner Lysholm Knee Scoring Scale (Özbek et al [11]) was a scale initially designed for patients with anterior cruciate ligament and meniscal injuries. This system consists of eight items (limp, pain, locking, stair climbing, support, instability, swelling, and squatting) and is scored on a scale of 0 to 100, with higher scores indicating fewer symptoms and higher levels of functioning.

The Olerud-Molander Ankle Score

The Olerud-Molander ankle score is also a valid, joint-specific assessment instrument that looks at the functional outcome of the ankle following a fracture. It evaluates nine functional components, pain, stiffness, swelling, daily activities, and other, more specific activities, for a maximum of 100 points. A 15-point difference is considered clinically important.

Discussion

Intramedullary nailing remains the primary treatment of choice for fixation of diaphyseal tibia fractures in adults. It comes with significant risks, with particularly high incidences of malrotation. This review sought to identify how to mitigate intraoperative risk by forming a systematic approach in the assessment of malrotation. Systematic exploration of the literature found that there is a lack of a systematic, standardized, or universal approach to managing tibia diaphyseal fractures with intramedullary nailing, and this is applicable to both - the intra- and postoperative period. 

With the lack of a universal approach to managing this cohort of patients, this article makes some suggestions as a framework to enable an initial pathway in creating standardization of care of these patients.

The aim of patient management should be to ‘Get It Right First Time’ (GIRFT), and the application of these evidence-based principles should be adhered to. Intraoperatively, the use of an external rotation tibial apparatus has been shown to be a reliable intraoperative assessment device and proven to statistically reduce the incidence of malrotation; the additional use of interlocking screws further confers extra longitudinal and rotational stability.

If clinical suspicion of postoperative rotational malalignment arises, CT imaging is the cross-sectional imaging of choice as it is reliable, quick and observer independent.

Patient-related functional outcomes should be assessed using tools that are validated and objective. The LEFS is already used commonly in practice, is validated, and addresses extensively all the daily activities a patient might face [7].

On review, it is apparent that there is still limited by level 1research that it is patient-focused and fully assesses the long-term functional impact on the various degrees of malrotation in post-tibial nailing surgery. Finally, it is recommended that further level-1 randomized controlled trial studies be conducted.

## Conclusions

As the most common long-bone fracture in the body, it is important that the management of tibia diaphyseal fractures are standardised and universal in their approach. With intramedullary nailing being the fixation of choice, it is important to identify that there is a high risk of malrotation and the severe consequences that this has on a patient's quality of life. This systematic review revealed there are gaps in the literature and in the management process of these patients, and suggested that a systematic approach using GIRFT, intraoperative assessment, validated assessment tools, and imaging postoperatively should be used to improve outcomes. Following the use of this framework, it is hoped that the incidence of malrotation post tibia intramedullary nailing will be reduced, however, it is acknowledged that more high-evidence studies need to be carried out and further done to optimise the care of these patients.
